# Editorial: Functional Adult Neurogenesis

**DOI:** 10.3389/fnins.2020.00885

**Published:** 2020-09-03

**Authors:** Yan Gu, Shaoyu Ge, Carlos P. Fitzsimons

**Affiliations:** ^1^Center of Stem Cell and Regenerative Medicine, Zhejiang University School of Medicine, Hangzhou, China; ^2^Department of Neurobiology and Behavior, Stony Brook University, Stony Brook, NY, United States; ^3^Faculty of Sciences, Swammerdam Institute for Life Sciences, University of Amsterdam, Amsterdam, Netherlands

**Keywords:** adult neurogenesis, hippocampus, olfactory bulb, neural stem/progenitor cells, newborn neurons

In the adult brains of most mammalian species, new neurons are continuously generated from neural stem/progenitor cells in discrete regions, such as the subgranular zone (SGZ) in the hippocampal dentate gyrus and the subventricular zone (SVZ) along the lateral cerebral ventricles. This process is generally termed adult neurogenesis, which is important for the survival of an individual in the natural environment. Accumulating studies have shown that the continuous adding of new neurons to the adult brain plays essential roles in relevant brain functions, such as spatial and fear memories, pattern separation, stress resilience, etc. Abnormalities in the generation or integration of new neurons are often associated with a various of disorders, such as mental disorders, stress disorders, epilepsy, etc. On the other hand, adult neurogenesis is regulated by a combination of molecular, cellular and circuitry mechanisms. This Research Topic collected several interesting and exciting new findings in the field of adult neurogenesis, regarding its functional implications.

During the development of newborn neurons in the adult brain, the initial morphogenic stage is critical for the survival, development, and functional integration of these newborn neurons. Ahamad et al. investigated the regulation of early morphogenesis of newborn neurons by a cellular metabolism-linked gene, Four and a half LIM domain 2 (FHL2). By using engineered viral vectors for genetic manipulation of FHL2 in the adult-born dentate granule neurons, they found that overexpression of FHL2 during early DGC development resulted in marked sprouting and branching of dendrites, while silencing of FHL2 increased dendritic length. These results suggest that FHL2 is an important regulator of early dendritic morphogenesis in adult-born dentate granule neurons, thus providing evidence for potential biological relevance of FHL2 in brain development and functions.

Tonic and phasic GABA signals regulate the development and integration of newborn neurons. Due to high level of ionic cotransporter NKCC1 expression in early-stage young neurons, GABAergic inputs initially provide depolarizing signals. As new neurons develop, accompanied by increasing expression of KCC2 and decreasing expression of NKCC1, GABA responses transit to hyperpolarizing signals. Gómez-Correa and Zepeda chronically administrated NKCC1 blocker bumetanide to young-adult rats, and found that the number of DCX-positive young neurons decreased, associated with altered morphological development of these newborn neurons. However, the animals' behavior was not affected in contextual fear conditioning and open field tests.

Evidence has shown that neurogenesis declines in the aging brain. Some of the most interesting questions arising from this observation are, how adult neurogenesis is affected by the microenvironment in the aging brain, and how adult neurogenesis may benefit the physiological functions of the aging brain. Trinchero et al. provided two studies related to adult neurogenesis in the aging brain. Their first study used whole-cell recordings in developing granule cells to characterize the time course of functional integration of adult-born granule neurons in aging mice, and found a later onset of functional excitatory synaptogenesis in aging mice than in young adult mice. Enriched environment significantly facilitated functional integration of newborn neurons in aging mice, indicating an experience-dependent structural plasticity and functional integration of newborn neurons in the aging brain. A second study from the same group showed long-term exercise accelerated the development of adult-generated dentate granule neurons. The accumulation of rapidly integrated newborn neurons generated under exercise are likely beneficial to hippocampus-dependent cognitive functions, possibly rejuvenating the hippocampal neural network in aging animals. These observations highlight how physical exercise could be a beneficial intervention to improve cognition in aging.

Early life stress affects the development of hippocampal neural circuits and postnatal behaviors. In a study by Daun et al., the authors utilized a maternal and social deprivation (MSD) model to investigate the effects of early life stress on neural stem cells and neurogenesis in the adult brain. They found that early life MSD enhanced neurogenesis not only in the dentate gyrus of the hippocampus, but also in the amygdala, such that the animals exposed to early life MSD exhibited a reduction in amygdala/hippocampus-dependent fear memory. This suggests that early life stress during a stress-hyporesponsive period may benefit the resilience to stress in adulthood.

Schizophrenia is a complex and serious mental disorder, and patients with schizophrenia are characterized by psychological hallucinations, deregulated emotionality, and cognitive impairment. Evidence indicated that postnatal neurogenesis in the hippocampus is profoundly impaired in schizophrenic individuals. As an extension of embryonic and early postnatal neurogenesis, adult neurogenesis in the hippocampus is susceptible to factors that are related to schizophrenia. Previous studies have shown that deficiency in schizophrenia-risk gene DISC1 results in deficits in the development of newborn neurons in the dentate gyrus and aberrant adult neurogenesis. Sheu et al. used a rodent model of schizophrenia through maternal immune activation of poly (I:C) injection, and found a delayed onset of schizophrenia-like pathology and the severity of the symptoms positively correlated with the aberrant dendritic phenotypes preferentially at 9-week-old of age for the animals. Temporal suppression of aberrant neurogenesis during such critical time period ameliorated the emergence of schizophrenia-like symptoms. These findings strongly suggest the aberrant dendritic growth of postnatal neurogenesis during a critical time window of development is essential for the pathophysiological progression of schizophrenia-like symptoms.

Resent observations have indicated that mating behavior may affect neurogenesis in the adult brain. In a study by Portillo et al., the authors investigated the effect of paced mating on adult neurogenesis in the olfactory bulb in female rats. They observed a significant increase in the percentage of new neurons in the granular and glomerular layers of the accessory olfactory bulb and granular layer of the main olfactory bulb in females that mated in four sessions, which paced sexual interaction, suggesting that repeated paced mating increases the percentage of new neurons that survive in the olfactory bulb of female rats.

The study of adult neurogenesis after injuries that affected the central nervous system has led to interesting observations suggesting that utilizing newly generated new neurons after injury may provide potential novel strategies for the functional recovery of impaired regions. The endogenous spinal cord ependymal cells, which form the central canal, represent a repair cell source in treating spinal cord injury. A study from Wang et al. showed that BAF45D, a member of the Brg1/Brm-associated factor (BAF) chromatin remodeling complex, is expressed in spinal cord ependymal cells, neurons, and oligodendrocytes but not astrocytes in rat spinal cord. After injury, the structure of central canal was disrupted and the BAF45D-positive spinal cord ependymal cell-derivatives were decreased. This study further highlighted the decreased expression of BAF45D in spinal cord ependymal cells in injured spinal cord, and the potential role of BAF45D downregulation in development of neuronal lesion after spinal cord injury. Their findings provided further understanding of the structural and biological roles of BAF45D in spinal cord ependymal cells after injury, and provided a potential target for spinal cord injury therapy via the manipulation of spinal cord ependymal cells.

Altogether, the articles included in this special Research Topic have identified novel mechanisms underlying the regulation of the generation, development, integration, and functions of newborn neurons in a variety of areas in the adult central nervous system, and provide meaningful insights for our understanding of functional neurogenesis in the adult nervous system.

## Author Contributions

YG, SG, and CPF wrote and edited the manuscript. All authors contributed to the article and approved the submitted version.

## Conflict of Interest

The authors declare that the research was conducted in the absence of any commercial or financial relationships that could be construed as a potential conflict of interest.

